# Exploring red cell distribution width as a biomarker for treatment efficacy in home mechanical ventilation

**DOI:** 10.1186/s12890-022-01916-0

**Published:** 2022-03-30

**Authors:** Luca Valko, Szabolcs Baglyas, Eszter Podmaniczky, Zoltan Prohaszka, Janos Gal, Andras Lorx

**Affiliations:** 1 Department of Anaesthesiology and Intensive Therapy, Home Mechanical Ventilation Program, Semmelweis University, 1082 Üllői út 78B, Budapest, Hungary; 2 Department of Internal Medicine and Hematology, Research Laboratory, Semmelweis University, 1428 POB2, Budapest, Hungary

**Keywords:** Biological marker, Chronic respiratory failure, Home mechanical ventilation, Red cell distribution width, Treatment efficacy

## Abstract

**Background:**

With the growing practice of home mechanical ventilation, there is a need to identify biological markers for adequate follow-up. Red cell distribution width (RDW) is a promising candidate because it is convenient, objective and may reflect treatment effect over a long period of time. The aim of this study was to explore the possible role of RDW as a marker for home mechanical ventilation in real-life, unselected chronic respiratory patient populations.

**Methods:**

First, we identified characteristic RDW values for mixed case, unselected chronic respiratory failure and home mechanical ventilated patients through retrospective review within our institutional database. Next, we conducted a prospective observational study to identify RDW changes during the first six months of optimized home mechanical ventilation treatment. Adult patients starting home mechanical ventilation were included. Factors affecting RDW change during the first 6 months of treatment were analysed.

**Results:**

RDW was elevated in both chronic respiratory failure and home mechanical ventilation patients compared to healthy individuals in the retrospective review. In the prospective study of 70 patients, we found that 55.4% of patients starting home mechanical ventilation have abnormal RDW values which are reduced from 14.7 (IQR = 13.2–16.2)% to 13.5 (IQR = 13.1–14.6)% during the first 6 months of HMV treatment (*p* < 0.001). RDW improvement correlates with improvement in self-reported health-related quality of life and sleepiness scale scores, as well as physical functional status during the same time frame. RDW proved to be a comparable marker to other parameters traditionally used to evaluate treatment efficacy.

**Conclusions:**

RDW is elevated in chronic respiratory failure patients and is significantly reduced in the first six months of optimized home mechanical ventilation. Although further research is needed to verify if RDW change reflects outcome and how comorbidities influence RDW values, our results suggest that RDW is a promising marker of home mechanical ventilation efficacy.

*Trial registration* This study was approved by and registered at the ethics committee of Semmelweis University (TUKEB 250/2017 and TUKEB 250-1/2017, 20th of December 2017 and 1st of October 2019).

## Background

Home mechanical ventilation (HMV) is increasingly used in several conditions of chronic respiratory failure (CRF) resulting in enhanced survival, reduced costs and improved health-related quality of life (HRQL) [1–4]. Guidelines have focused on patient selection and treatment initiation, but there is less data on how patients should be managed in the long term, and what markers are indicative of efficient therapy and increased chance of survival. Monitoring and intermittent analysis of vital signs and respiratory parameters recorded by ventilators are possible tools for evaluating treatment efficacy, but might pose a cumbersome method and are difficult to generalize [5]. Blood gas parameters are regarded as treatment goals, but sampling is painful and results may vary based on sampling time and method, resulting in unreliable detection of insufficient support and dubious outcome prediction [2, 6, 7]. Self-reported parameters such as the Epworth Sleepiness Scale (ESS) and the Severe Respiratory Insufficiency Questionnaire (SRI) might be indicative of long-term outcome, but results are subjective and variable [8–11]. Laboratory biomarkers, especially ones able to reflect persistent effects over a period of time akin to HbA1c measurements in diabetic patients, pose an attractive alternative means of long-term monitoring, but so far no targets have been identified to evaluate treatment efficacy and to predict outcome in HMV patients.

Red cell distribution width (RDW), the variability in size of red blood cells traditionally used to differentiate anaemias, has gained wide recognition in recent years as a chronic disease prognostic marker [12]. RDW and its association to poor outcome has been studied in several disease groups and populations (e.g.: cardiovascular diseases, cancer, community acquired pneumonia, venous thrombosis and in the general population), though it is unclear whether RDW is an independent predictor or rather a common marker of different pathways (inflammation, oxidative stress, aging, nutrition, erythropoiesis) affecting outcome [13–18].

As erythropoiesis is directly influenced by hypoxic stimuli, it is reasonable to expect that hypoxic CRF patients would have elevated RDW as a result of increased production of new, larger erythrocytes prompted by episodes of hypoxia. This mechanism is referred to as the erythropoietin risk-pathway model and has been credited in producing elevated RDW values in several conditions that might require HMV, such as chronic obstructive pulmonary disease (COPD), muscular dystrophies and quadriplegia [12, 19]. Additionally, several other pathological pathways might contribute to abnormal RDW values in chronic respiratory failure such as inflammation, metabolic disturbances and nutritional issues.

The aim of this study was to observe characteristic RDW alterations in different chronic respiratory failure populations and the RDW changes associated with initiation of HMV treatment in a real-life, mixed case chronic respiratory patient population.

## Methods

### Study design

We first identified characteristic RDW values for mixed case, unselected CRF and stable HMV patients compared to healthy individuals through retrospective review within our institutional database.

Next, we conducted a prospective observational study to evaluate RDW changes in an unselected, real-life CRF population starting optimized HMV. Primary endpoint was RDW change at six months after starting HMV. Secondary endpoints were factors associated with RDW change during the first six months of HMV treatment.

### Patients

The retrospective review included patients diagnosed with CRF referred to the Semmelweis University Home Mechanical Ventilation Program from 2014 to 2018, with no history of previous HMV, as well as patients already established on HMV and compared them to a sex- and age-matched healthy population. For CRF and HMV patients, laboratory data acquired during stable condition (defined as lack of respiratory treatment alteration because of an acute condition) were used. Healthy volunteers were employees scheduled for routine laboratory work up as a part of occupational health appointments at our department.

The prospective study enrolled patients starting HMV through the Semmelweis University Home Mechanical Ventilation Program from January 2018 to June 2020. Clinical and laboratory data were measured at baseline, then one, three and six months after starting treatment. Inclusion criteria were > 18 years of age, diagnosed CRF and HMV treatment of at least 6 h/day. Exclusion criteria were previous HMV treatment, noncompliance with HMV and haematological disorders. Recruitment lasted from January of 2018 to June of 2020, with the last follow-up completed in December of 2020.

Written informed consent was obtained from all patients included in the study. The study was approved by the ethics committee of Semmelweis University (TUKEB 250/2017 and TUKEB 250-1/2017).

### HMV treatment

HMV was supplied by A40 or Trilogy 100 ventilators (Koninklijke Philips N.V., Amsterdam) and AIRcon or HC150 humidifiers (WILAmed GmbH, Kammerstein; Fishel Paykel Healthcare, Auckland) in pressure controlled, volume targeted mode through a noninvasive interface if possible or invasive interface if noninvasive ventilation was contraindicated/not feasible. Inspiratory time was aimed at 25% (patients with obstructive lung function characteristics) and 30% (all other patients), then further tuned for patient comfort. Supplementary O_2_ was prescribed if paO_2_ was below 60 mmHg. Treatment goals for HMV were based on the German HMV guideline and included > 80% compliance with a minimum of 6 h daily ventilator use, normalization of paCO_2_ and paO_2_ levels [20, 21]. Respiratory secretion management was achieved by cough assisting device (CoughAssist T70, Koninklijke Philips N.V., Amsterdam) and/or tracheal suction devices if peak expiratory flows were below 2.5 L/s [22].

During the course of the study period, patients received personalized, optimized HMV therapy as defined by monthly or bimonthly follow-up by a physician trained in HMV. Follow-up appointments involved equipment checks, treatment goal verification, ventilator waveform analysis and vital sign checks with consequent adjustments of HMV therapy, if necessary.

### Data collected

In the retrospective analysis, demographic data (age, sex), treatment characteristics (diagnosis/indication for HMV, interface type, HMV duration, O_2_ supplementation need) and laboratory parameters (RDW, hemoglobin [HGB], hematocrit [HCT], red blood cell count [RBC], mean cell volume [MCV], mean cell hemoglobin [MCH], mean cell hemoglobin concentration [MCHC], white blood cell count [WBC], platelet count [PLT], mean platelet volume [MPV], C-reactive protein [CRP]) were collected using our institutional electronic medical record system.

In the prospective study, demographic data (age, sex, body mass index [BMI]), clinical parameters (6-min walking distance [6MWD], ESS, SRI summary and respiratory complaint scores [SRI-SS and SRI-RC]) (HRQL markers that are more significantly altered after initiation of HMV) [23], treatment characteristics and ventilatory parameters (indication for HMV, referral type, interface, HMV duration, daily ventilation, O_2_ supplementation, inspiratory and expiratory time ratio [Ti/Tt], inspiratory positive airway pressure [IPAP], expiratory positive airway pressure [EPAP], respiratory rate [RR], tidal volume [VT], patient triggered breath percentage [PTB]), blood gas values (pH, partial pressure of oxygen [paO_2_], partial pressure of carbon-dioxide [paCO_2_], bicarbonate [HCO_3_]), pulmonary function test (PFT) parameters (forced vital capacity percentage [FVC%], forced 1 s expiratory volume percentage [FEV1%], FEV1/FVC%, peak expiratory flow percentage [PEF%]) and laboratory parameters (RDW, HGB, HCT, RBC, MCV, MCH, MCHC, WBC, PLT, MPV, CRP) were collected.

PFTs were performed with the Piston PinkFlow meter (Piston Ltd, Budapest, Hungary). Arterial blood gas sampling was performed minimum 15 min after discontinuing ventilation and/or oxygen supplementation, unless patients were ventilator dependent. Laboratory parameters were determined from standard venous blood draw. The SRI and ESS were used for self-reported parameters [24]. Indications for HMV included COPD, restrictive chest wall disease (RCWD), obesity hypoventilation syndrome (OHS), slowly progressing neuromuscular disease (NMD), amyotrophic lateral sclerosis (ALS), other pulmonary diseases (PD) or spinal cord transection (SCT). RDW was measured as part of an automated complete blood count on a Sysmex XN-1000 (Sysmex, Kobe, Japan) haematology analyser and reported as the coefficient of variation of the mean red blood cell volume. The same laboratory method was used in both the retrospective and prospective studies as to ensure comparability of RDW values [25]. Reference range was defined as 11.5–14.5%

### Statistical analysis

Variables are shown as n (%) and median (interquartile range). Normality was tested with the Kolmogorov–Smirnov test. Comparison between the parameters of the three groups in the retrospective analysis was performed using the Kruskal–Wallis ANOVA by Ranks test for continuous, and the Pearson Chi-square test for categorical variables. Kruskal–Wallis ANOVA was performed with multiple comparison post-tests for pairwise comparison for RDW values. Mann–Whitney U test was used when comparing only two groups. Propensity matching of healthy subjects was performed manually based on age and sex.

Sample size calculation for the prospective study was performed based on the characteristic values and effect size of the CRF and HMV populations of the retrospective review using a dependent sample t test as a statistical design, power of 0.80 and α = 0.05 as well as an estimated dropout rate of 15%. (Mu1: 15.2 Mu2: 14.0 SD1: 3.01 SD2: 1.63, power: 0.8, Rho: 0.1).

In the prospective study, the Wilcoxon matched pair test was used to compare continuous variables at the baseline and 6-month time points with post-hoc subgroup analysis for diagnostic groups. Ratio of patients with abnormal RDW values was calculated using the Pearson Chi-square test. Time related changes across all time points were analysed with Friedman ANOVA. The relationship between baseline RDW values and other variables of clinical interest as well as RDW changes and other variables of clinical interest was evaluated using Spearman Rank correlation. Significant outcome measure correlations were verified by Kruskal Wallis ANOVA by Ranks with grouping based on the presence of clinically relevant RDW improvement (defined as clinically relevant improvement of 0.5% from baseline, based on previously published data) [26]. Differences in baseline RDW values by group were also analysed with Mann–Whitney U test and Kruskal Wallis ANOVA by Ranks (sex, interface type, referral type, HMV indication). Logistic regression was used to identify variables closely related to clinically relevant improvement in RDW values including data from each study time point. After univariate analysis of possible predictors (age, sex, BMI haemoglobin, HMV duration, HMV indication, interface type, referral type, paO_2_, paCO_2_), all significant variables and previously identified possible prognostic factors of RDW (age, sex, haemoglobin value) were entered into a multivariate logistic analysis [27]. Receiver operating characteristic curves were created using treatment time points as the reference standard (before treatment and at 6 months of treatment) and RDW and other traditionally used treatment efficacy markers (paCO_2_, HCO_3_, SRI-SS, SRI-RC and ESS values) as continuous predictors. Predictors were compared based on their ability to best discriminate between non-treated and treated patients based on their AUC values.

Missing data was not used in the calculations. Statistical analyses were performed using STATISTICA 10.0 (StatSoft Inc, Tulsa, OK, USA) and Statistical Package for Social Sciences (IBM Corporation, Armonk, NY, USA) 25.0.

## Results

### Characteristic RDW values by patient groups

The retrospective review included 191 patients (79 CRF patients, 83 established HMV patients and 29 healthy volunteers) (see Table 1).

**Table 1:** Patient characteristics of the retrospective review

	CRF patients (n = 79)	HMV patients (n = 83)	healthy patients (n = 29)	*p*-value
**Demographic data**				
Age (years)	53 (41–62)	55 (42–69)	53 (41–64)	*p* = 0.537
**Sex**				
Female	20 (25.3%)	22 (26.5%)	7 (24.1%)	*p* = 0.965
Male	59 (74.7%)	61 (73.5%)	22 (75.8%)	
**Diagnosis/HMV indication**				
COPD	8 (10.1%)	13 (15.7%)		*p* = 1.000
RCDW	5 (6.3%)	4 (4.8%)		
OHS	26 (32.9%)	26 (31.3%)		
NMD	19 (24.1%)	20 (24.1%)		
ALS	14 (17.7%)	12 (14.5%)		
PD	2 (2.5%)	3 (3.6%)		
SCT	5 (6.3%)	5 (6.0%)		
**Interface**				
Noninvasive		68 (81.9%)		
Invasive		15 (18.1%)		
HMV duration (months)		6 (4–27)		
O_2_ supplementation need				
No	45 (57.0%)	47 (56.6%)		*p* = 1.000
Yes	34 (43.0%)	36 (43.4%)		
**Laboratory parameters**				
RDW (%)	14.5 (13.2–15.6)	13.9 (13.2–15.3)	12.8 (12.3–13.6)	***p* < 0.001**
HGB (g/L)	130 (107–149)	137 (121–148)	141 (133–151)	*p* = 0.051
HCT (L/L)	0.39 (0.34–0.46)	0.4 (0.37–0.45)	0.41 (0.39–0.44)	*p* = 0.503
RBC (Tera/L)	4.42 (3.82–5.17)	4.67 (4.18–5.02)	4.7 (4.41–4.96)	*p* = 0.555
MCV (fL)	87.2 (85–89.5)	87.8 (85.5–90.8)	87.9 (85.6–89.8)	*p* = 0.653
MCH (pg)	29 (27.4–29.8)	29.2 (28.4–30.5)	29.6 (28.9–30.7)	***p* = 0.011**
MCHC (g/L)	327 (315–337)	333 (325–341)	342 (334–347)	***p* < 0.001**
WBC (Giga/L)	8.33 (6.77–10.86)	8.71 (7.12–10.77)	6.33 (5.7–7.65)	***p* < 0.001**
PLT (Giga/L)	261 (215–346)	248 (218–306)	255 (231–282)	*p* = 0.317
MPV (fL)	10.35 (9.1–11.1)	10.8 (9.9–11.5)	10.3 (10–10.8)	***p* = 0.009**
CRP (mg/L)	7.2 (2.5–19.1)	6.35 (2.7–11.8)	2.7 (1.6–10.6)	*p* = 0.065

Significant differences are marked with bold font

Data is presented as n (%) and median (IQR). Comparison between the parameters of the three groups in the retrospective analysis was performed using the Kruskal–Wallis ANOVA by Ranks test for continuous, and the Pearson Chi-square test for categorical variables. Mann–Whitney U test was used when comparing two groups (such as in the case of O_2_ supplementation need and diagnosis differences). Significant differences are marked with bold font

*ALS* amyotrophic lateral sclerosis, *COPD* chronic obstructive pulmonary disease, *CRF* chronic respiratory failure, *CRP* C-reactive protein, *HCT* haematocrit, *HGB* haemoglobin, *HMW* home mechanical ventilation, *MCH* mean cell haemoglobin, *MCHC* mean cell haemoglobin concentration, *MCV* mean cell volume, *MPV* mean platelet volume, *NMD* neuromuscular disease, *OHS* obesity hypoventilation syndrome, *PD* pulmonary disease, *PLT* platelet, *RBC* red blood cell, *RCDW* restrictive chest wall disease, *RDW* red cell distribution width, *SCT* spinal cord transection, *WBC* white blood cell

**Fig. 1:**
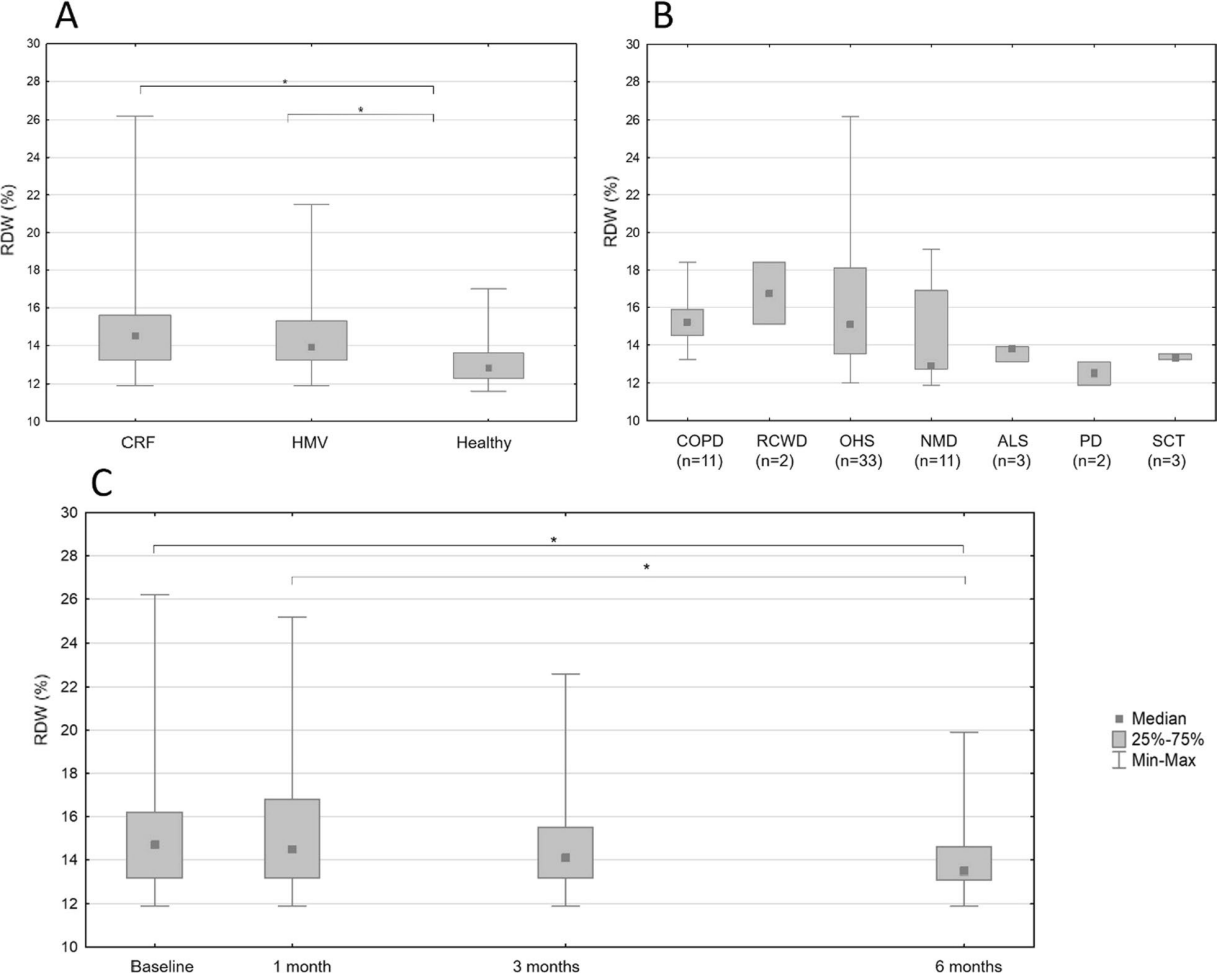
RDW values for different patient populations. **A** RDW values of different patient groups measured in the retrospective review: Boxplot of RDW values of CRF and HMV patient groups compared to healthy volunteers. Kruskal Wallis ANOVA *p*-value is < .001 for the three groups, significant differences marked with an asterix are based on pairwise post-hoc testing. **B** Baseline RDW values by HMV indications measured in the prospective study: Boxplot of baseline RDW values of HMV patients grouped by diagnosis. **C** RDW values after initiation of HMV at different time points of the prospective study: Boxplot of RDW values at different time points of HMV treatment. Significant differences, based on Friedman ANOVA, are marked with an asterix (0 vs 6 months: *p* < 0.001; 1 vs 6 months: *p* = 0.002)

Post-hoc analysis showed that there was a significant difference in RDW values in both ventilation naive CRF patients [14.5 (13.2–15.6)%] and in HMV patients [13.9 (13.2–15.3)%] compared to healthy individuals [12.8 (12.3–13.6)%] (*p* < 0.001 and *p* = 0.001 respectively). A tendency for reduced RDW values could be observed in HMV patients compared to CRF patients (see Fig. 1A).

### Prospective change in RDW during optimized HMV

70 patients were enrolled in the prospective study based on the results of the sample size calculation, 65 patients completed the study (3 died and 2 were excluded because of noncompliance). Median age for the study group was 54 (IQR = 47–63), 24.6% were female. Median BMI was 38.2 (IQR = 25.5–46.7) kg/m^2^. 12.3% of patients used invasive and 87.7% noninvasive ventilation. At baseline, 50.8% of patients required supplementary O_2_. Indications for HMV were COPD in 11 (16.9%), RCDW in 2 (3.1%), OHS in 33 (50.8%), NMD in 11 (16.9%), ALS in 3 (3.1%), PD in 2 (3.1%) and SCT in 3 (3.1%) patients. 36 patients (55.4%) were recruited to the HMV program through chronic care referral, while 29 patients (44.6%) were referred to the program after an acute exacerbation of their underlying disease (hospital referral).

Recorded parameters at baseline and at six months are listed in Table 2. Significant improvements were observed in six-minute walking distance (6MWD), ESS, SRI Summary Score (SRI-SS), blood gas values and PFT parameters with no relevant changes in ventilatory parameters.

**Table 2:** Clinical parameters of the prospective observational study

	Baseline	6 months	
**Clinical parameters**			
BMI (kg/m^2^)	38.2 (25.5–46.7)	39.9 (28.7–46.6)	*p* = 0.125
6MWD (m)	155 (0–276)	265.3 (1–359)	***p* < 0.001**
ESS (points)	9 (6–14)	3 (2–5.5)	***p* < 0.001**
SRI-SS (points)	57.2 (49.6–72.1)	68.9 (56.4–84.6)	***p* < 0.001**
**Ventilatory parameters**			
Daily ventilation (h)	8.3 (7,0–9.7)	7.1 (6–8.5)	***p* < 0.001**
O_2_ supplementation (L/min)	1.5 (0–3)	0 (0;2)	***p* < 0.001**
( No/yes)	32/33 (49.2%/50.8%)	36/29 (55.4/44.6%)	
Ti/Tt (s/s)	33.8 (31.3–36.2)	33.9 (31.5–37.1)	*p* = 0.385
IPAP (cmH_2_O)	20.6 (17.7–25.6)	20.0 (17.0–22.9)	***p* < 0.001**
EPAP (cmH_2_O)	9.9 (7.9–12.9)	9.9 (7.9–12.9)	*p* = 0.955
RR (/min)	15.7 (14.4–17.2)	15.6 (14.3–16.7)	*p* = 0.726
VT (mL)	623 (516–728)	625 (517–722)	*p* = 0.878
PTB (%)	55.9 (36–79)	60.7 (41.5–78.7)	*p* = 0.647
**Blood gas values**			
pH	7.41 (7.39–7.44)	7.4 (7.38–7.43)	*p* = 0.278
paO_2_ (mmHg)	68.9 (58.5–83.2)	78.2 (69.8–87.2)	***p* = 0.002**
paCO_2_(mmHg)	45 (37.0–52.3)	39.5 (36.4–44.3)	***p* < 0.001**
HCO_3_ (mmol/L)	29.1 (24.3–31.4)	25.0 (22.6–27.2)	***p* < 0.001**
**Pulmonary function test parameters**			
FVC% (%)	59 (41–72)	65 (48–80)	***p* < 0.001**
FEV1% (%)	50 (32–71)	57 (36–76)	***p* < 0.001**
FEV1/FVC% (%)	96 (88–108)	97 (84–105)	*p* = 0.314
PEF% (%)	48 (31–64)	56 (35–70)	***p* = 0.009**
**Laboratory parameters**			
RDW (%)	14.7 (13.2–16.2)	13.5 (13.1–14.6)	***p* < 0.001**
HGB (g/L)	142 (123–155)	142 (130–151)	*p* = 0.119
HCT (L/L)	0.43 (0.38–0.47)	0.42 (0.4–0.45)	*p* = 0.859
RBC (Tera/L)	4.98 (4.33–5.39)	4.82 (4.37–5.23)	*p* = 0.925
MCV (fL)	86.8 (85.1–89.2)	87.3 (85.5–90.3)	*p* = 0.664
MCH (pg)	28.6 (27.1–29.9)	29.2 (28.3–30.3)	***p* < 0.001**
MCHC (g/L)	327 (310–339)	334 (327–340)	***p* < 0.001**
WBC (Giga/L)	8.0 (6.8–10.5)	8.4 (7.1–9.9)	*p* = 0.769
PLT (Giga/L)	257 (211–319)	257 (219–301)	*p* = 0.354
MPV (fL)	10.8 (9.9–11.2)	10.8 (10–11.3)	*p* = 0.070
CRP (mg/L)	10.0 (4.5–20.3)	6.7 (3.8–11.9)	***p* = 0.002**

Significant differences are marked with bold font

Wilcoxon matched pair test for continuous variables. Significant differences are marked with bold font. For false discovery rate correction Benjamini–Hochberg method was used and all bold shaped p-values remained significant after this correction. Pulmonary function parameters are listed as percentage of expected value based on weight, height and age according to Quanjer 1993

*6MWD* six-minute walking distance, *BMI* body mass index, *CRP* C-reactive protein, *EPAP* expiratory positive airway pressure, *ESS* epworth sleepiness scale, *FEV1%* forced 1 s expiratory volume percentage, *FVC%* forced vital capacity percentage, *HCO*_*3*_ bicarbonate, *HCT* haematocrit, *HGB* haemoglobin, *HMW* home mechanical ventilation, *IPAP* inspiratory positive airway pressure, *MCH* mean cell haemoglobin, *MCHC* mean cell haemoglobin concentration, *MCV* mean cell volume, *MPV* mean platelet volume, *paCO*_*2*_ partial pressure of carbon-dioxide, *paO*_*2*_ partial pressure of oxygen, *PEF%* peak expiratory flow percentage, *PLT* platelet, *PTB* patient triggered breath percentage, *RBC* red blood cell, *RDW* red cell distribution width, *RR* respiratory rate, *SRI-SS* severe respiratory insufficiency questionnaire summary score, *Ti/Tt* inspiratory and expiratory time ratio, *VT* tidal volume, *WBC* white blood cell

At baseline 36 patients (55.4%) had abnormal RDW values, compared to 16 (24.6%) at the 6-month mark (*p* < 0.01). 43 patients (66.2%) showed diminished RDW values compared to their baseline at the end of the study period. Median change in RDW was −0.4% (IQR: −2.2–0.2).

RDW values decreased significantly during six-month follow-up from 14.7 (13.2–16.2) to 13.5 (13.1–14.6) % (*p* = 0.009) (see Fig. 1C).

Baseline RDW values showed significant correlations with BMI (R = 0.415, *p* < 0.001), pO_2_ (R = 0.366, *p* = 0.003), forced 1 s expiratory volume/forced vital capacity percentage (FEV1/FVC%) (R = −0.283, *p* = 0.022), HGB (R = −0.262 *p* = 0.035), PLT (R = 0.261, *p* = 0.035), CRP (R = 0.572, *p* < 0.001) and SRI-RC (R = −0.316, *p* = 0.010). There was no difference in baseline RDW based on sex (*p* = 0.190) or interface type (*p* = 0.646), but there was a significant baseline RDW difference in patients based on referral type [15.73 (11.90–26.13) % for hospital referral and 13.52 (11.90–21.20)% for chronic care referral *p* < 0.001] and indication for HMV (*p* = 0.050) (see Fig. 1B).

**Fig. 2:**
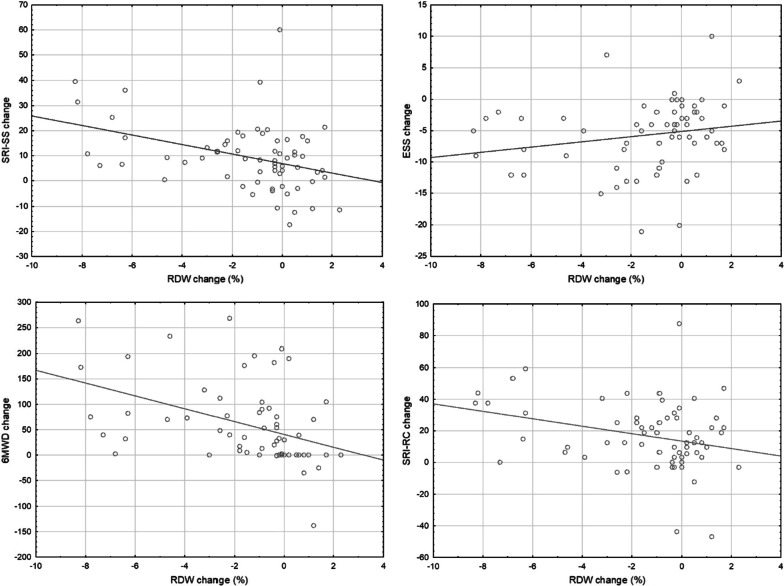
Scatter plot diagrams for RDW change correlations with other parameters at six months of home mechanical ventilation treatment. 6MWD: six-minute walking distance, ESS: Epworth Sleepiness Scale, RDW: red cell distribution width, SRI-RC: Severe Respiratory Insufficiency Questionnaire Respiratory Complaints, SRI-SS: Severe Respiratory Insufficiency Questionnaire Summary Score

Furthermore, we found that RDW change correlates significantly with change in SRI-SS and ESS values (R = −0.364, *p* < 0.01 and R = −0.295, *p* = 0.02 respectively), as well as change in 6MWD values (R = −0.542, *p* < 0.01) during the first six months of HMV (see Fig. 2). Change of RDW values did not show a significant correlation with change in SRI-RC values (R = −0.226, *p* = 0.07). Patients with clinically relevant improvement in RDW showed larger improvements in SRI-SS, ESS and 6MWT values [10.9 (5.0–17.7) vs. 4.4 (−2.7–10.2), *p* = 0.011; −6 (−11 to −3.5) vs. −3 (−6.8 to −1), *p* = 0.029; and 60 (18.5–108) m vs. 0 (0–15) m *p* < 0.001 respectively).

RDW change was significant for COPD (*p* = 0.021) and OHS groups (*p* < 0.001). There was a tendency for reduction in RDW values for RCWD patients, although the patient sample size in this group was too small to make significant observations (*p* = 0.180). Other diagnoses had no significant change in RDW values during the course of the first 6-months of HMV treatment, although limited patient numbers also might have played a role.

Univariate logistic regression found HMV duration, RCDW and OHS diagnosis to be significant predictors of RDW improvement. Multivariate logistic regression was performed with these variables as well as previously cited prognostic factors (age, sex and haemoglobin value). After adjusting for these factors, multivariate logistic regression revealed that RDW improvement is dependent on duration of HMV, with an odds ratio of 1.65 per month. RDW improvement is independent of sex, age and baseline haemoglobin in our model. Improvement is dependent on diagnosis, with RCWD and OHS associated with the biggest chance of improvement in RDW values (see Table 3).

**Table 3:** Predictors of RDW improvement after HMV initiation

Variable	Univariate OR (95% CI)	Walds Chi^2^ *p*-value	Multivariate OR (95% CI)	Walds Chi^2^ *p*-value
HMV duration (months)	1.51 (1.34–1.72)	*p* < 0.001	1.65 (1.42–1.91)	*p* < 0.001
**Indication for HMV**				
RCWD	30.00 (2.22–405.98)	*p* = 0.011	66.86 (3.63–1232.65)	*p* = 0.005
OHS	9.84 (1.22–79.18)	*p* = 0.032	13.04 (1.35–132.73)	*p* = 0.027

Logistic regression was used to identify variables closely related to improvement in RDW values (defined as clinically relevant improvement of 0.5% from baseline) including data from each study time point (n = 262). After univariate analysis of possible predictors (age, sex, haemoglobin value, HMV duration, HMV indication, interface type, referral type, paO_2_, paCO_2_), all significant variables and previously cited possible prognostic factors (age, sex, haemoglobin value) of RDW were entered into multivariate logistic analysis

*OR* odds ratio, *CI* confidence interval, *HMV* home mechanical ventilation, *OHS* obesity hypoventilation syndrome, *RCDW* restrictive chest wall disease

**Fig. 3:**
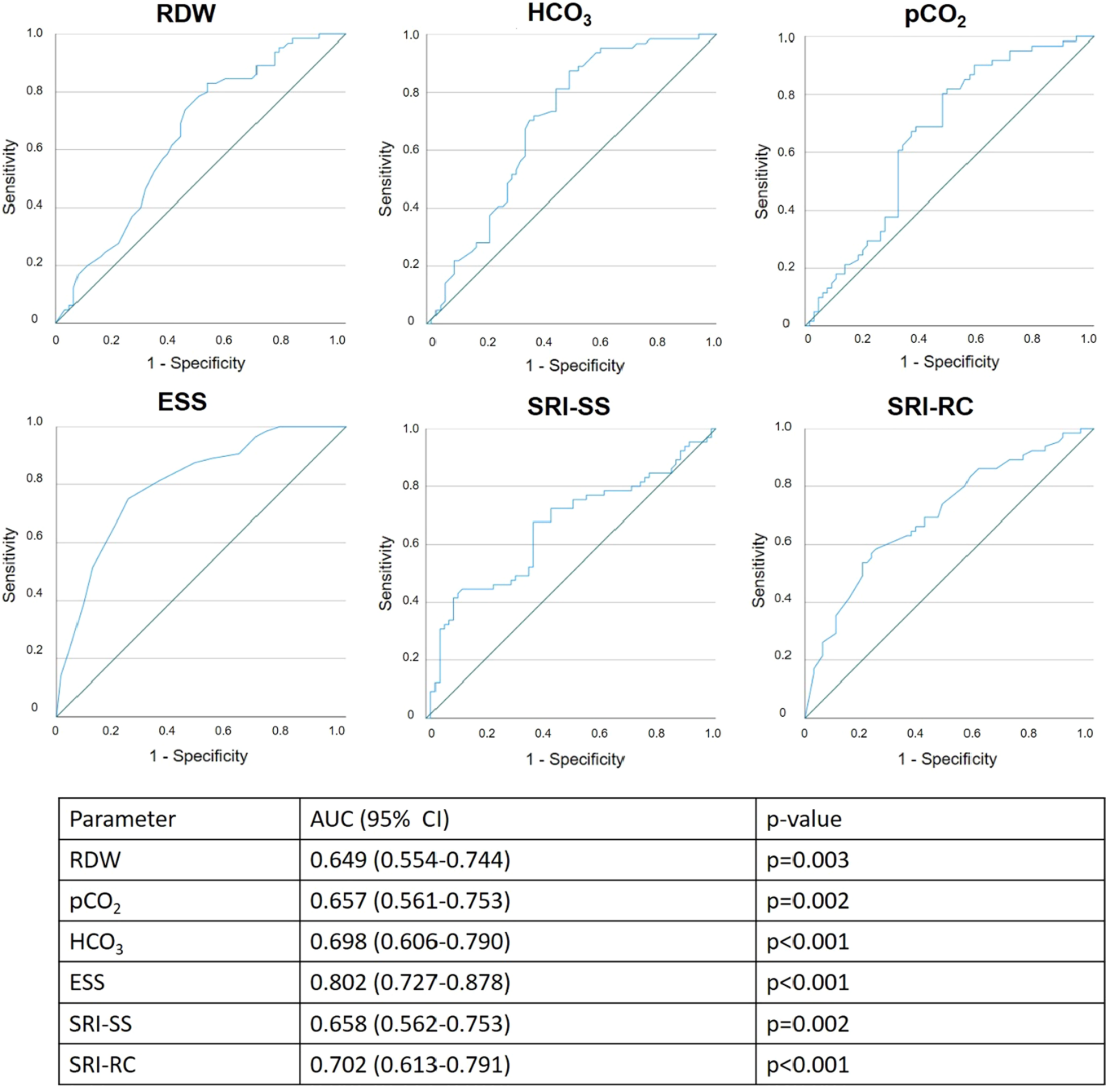
Receiver operating characteristic curves against 50% reference lines for different potential treatment efficacy markers of home mechanical ventilation. Receiver operating characteristic curves were created using treatment time points as the reference standard (before treatment and at 6 months of treatment) and RDW and other traditionally used treatment efficacy markers (paCO_2_, HCO_3_, SRI-SS, SRI-RC and ESS values) as continuous predictors. Predictors were compared based on their ability to best discriminate between non-treated and treated patients based on their AUC values. ESS: Epworth Sleepiness Scale, HCO_3_: bicarbonate, paCO_2_: arterial partial pressure of carbon-dioxide, paO_2_: arterial partial pressure of oxygen, RDW: red cell distribution width, SRI-RC: Severe Respiratory Insufficiency Questionnaire Respiratory Complaints, SRI-SS: Severe Respiratory Insufficiency Questionnaire Summary Score

When comparing the ability of RDW values to identify the six-month time point after starting HMV treatment to other parameters traditionally used to evaluate HMV patients, RDW performed similarly to paCO_2_, HCO_3_ and SRI-SS values, although remained inferior to ESS and SRI-RC values (see Fig. 3).

## Discussion

This is the first study demonstrating that elevated RDW values show gradual reduction during optimized HMV treatment in an unselected population of CRF patients, parallel to HRQL and physical function improvement, suggesting that RDW might be indicative of outcome and is a useful marker in the HMV population.

Several chronic respiratory conditions have been described to result in elevated RDW values with associated poor outcomes, such as COPD and idiopathic pulmonary fibrosis [28–31]. It has also been reported that increased RDW values show a positive independent correlation with apnoea-hypopnea index (AHI), a marker of obstructive sleep apnoea (OSA) severity [32–35]. In addition, a pathome-wide analysis based on a medical claims database of 2 million patients showed that elevated RDW values were associated with several conditions that are potential indications of HMV, such as COPD, idiopathic pulmonary fibrosis, muscular dystrophy, myasthenia gravis and quadriplegia [12]. These previously published data are corroborated by the results of our prospective observational study, which found a median baseline RDW value of 14.7% (IQR: 13.2–16.2) in an unselected population of CRF patients, with COPD, RCWD and OHS patients (a population with OSA as a common comorbidity) showing frequent abnormal RDW values.

RDW as a possible treatment marker has only been studied in a few conditions before now. While RDW change has been proven to correlate with myocardial infarction treatment efficacy, data with pulmonary arterial hypertension patients is conflicting [36–38]. Data with possible implications for CRF patients is even more scarce: a study on OSA patients receiving CPAP therapy showed no change after one year despite ESS reduction while another study with smaller patient numbers found an increase in RDW values after six months [32, 39]. RDW changes have not been previously reported in CRF patients receiving long-term mechanical ventilation using bilevel settings. In our prospective study we found that during the first six months of optimized HMV treatment, which was verified by several improving clinical parameters in our cohort, median RDW values are reduced from 14.7 to 13.5% in an unselected CRF population.

Data showing greater improvement in self-reported HRQL and sleepiness scales, as well as physical functional status in the group with clinically relevant RDW improvement are promising, as these factors have been reported to be predictive of mortality in HMV, which suggests that RDW change may correspond with improving outcome [8, 40]. Additionally, the data suggesting that RDW improvement at six months of HMV treatment is comparably reliable as improvements in other parameters traditionally used to evaluate treatment efficacy, such as paCO_2_, ESS and SRI-SS, while ostensibly having the advantage of more objective, consistent and reliable sampling, makes RDW a promising candidate for future research.

Our study has several limitations, most importantly that it includes a heterogeneous patient population with high probability of other causes influencing RDW values. As this was a preliminary study evaluating the potential role of RDW as a treatment marker for HMV, we aimed to include a mixed-case patient population reflecting the real-life practice of HMV centres. Our results indicate that different patient groups have different RDW values, although we did not investigate whether specific comorbidities might play a role in this. Further studies should focus on the potential confounding roles of comorbidities, which patients are more prone to show elevated RDW values and what predictive role elevated RDW values play. The baseline RDW differences observed based on referral type in our study suggest that patients with hospital referrals (generally booked after an episode of acute exacerbation) might explain the increased RDW values and subsequent improvement we observed, as acute illness often entails blood transfusions, ischemic episodes and septic complications, which have all been reported to cause elevated RDW [41–43]. As previously reported, acute hypoxia-associated diseases cause RDW elevation with a maximum one month after onset of the condition and normalization about six months later [19]. Although we found similar RDW change kinetics after starting HMV treatment, referral type did not predict RDW improvement in our model. Additionally, as described in the previously referenced study, pleural effusion and pneumothorax is associated with large increases in RDW, while pneumonia leads to smaller RDW increments and most diseases of airway obstruction (asthma, COPD, OSA) do not prompt sudden increase in RDW values, suggesting that the values recorded in the hospital referral group in our prospective study were not the effect of the acute exacerbation precluding the referral but rather a marker of chronic disease severity (e.g. sicker patients were more likely to be recruited to the HMV program in association with an acute exacerbation) [19].

As an additional limitation, our study was not powered to establish definite differences between HMV indications, although our results suggest that different conditions are associated with different RDW kinetics during HMV treatment. As RDW change was independent of baseline paO_2_, it is probable that not only de facto hypoxia but ventilation disturbances in general might play a role in RDW changes in CRF patients. Diminishing hypercapnia related circulatory effects and fatigue as well as improving metabolic alterations may contribute to RDW change as a result of improvement in overall respiratory status [44–46]. This might help explain the robust RDW change we observed in OHS patients, despite previous data showing that OSA related RDW alterations did not respond to CPAP treatment. Other potential mechanisms of RDW reduction due to HMV treatment might include improving inflammatory and nutritional status, as evidenced by decreasing inflammatory values and improving functional status in our study, which correlate with RDW change.

Further research is needed to identify the predictive value of RDW values and their kinetics in this population, which disease groups are ideal for follow up with RDW measurement, how often RDW should be measured and whether improving or worsening RDW values correspond with outcome.

In conclusion, elevated RDW values in CRF patients show reduction during the first 6 months of optimized HMV treatment parallel to clinical improvements, suggesting that RDW is a useful marker in HMV patients.

## Data Availability

The deidentified datasets analyzed during the current study are available from the corresponding author on reasonable request.

## Abbreviations

6MWD: 6-Minute walking distance
AHI: Apnoea hypopnoea index
ALS: Amyotrophic lateral sclerosis
ANOVA: Analysis of variance
BMI: Body mass index
CI: Confidence interval
COPD: Chronic obstructive pulmonary disease
CRF: Chronic respiratory failure
CRP: C-reactive protein
EPAP: Expiratory positive airway pressure
ESS: Epworth Sleepiness Scale (ESS)
FEV1%: Forced 1 s expiratory volume percentage
FVC%: Forced vital capacity percentage
HCO_3_: Bicarbonate
HCT: Hematocrit
HGB: Hemoglobin
HMV: Home mechanical ventilation
HRQL: Health-related quality of life
IPAP: Inspiratory positive airway pressure
NMD: Neuromuscular disease
MCH: Mean cell hemoglobin
MCHC: Mean cell hemoglobin concentration
MCV: Mean cell volume
MPV: Mean platelet volume
OHS: Obesity hypoventilation syndrome
OR: Odds ratio,
OSA: Obstructive sleep apnoea
paCO_2_: Partial pressure of carbon-dioxide
paO_2_: Partial pressure of oxygen
PEF%: Peak expiratory flow percentage
PFT: Pulmonary function test
PD: Pulmonary disease
PLT: Platelet count
PTB: Patient triggered breath percentage
RBC: Red blood cell count
RCWD: Restrictive chest wall disease
RDW: Red cell distribution width
RR: Respiratory rate
SCT: Spinal cord transection
SRI: Severe Respiratory Insufficiency Questionnaire
SRI-RC: SRI respiratory complaint score
SRI-SS: SRI summary score
Ti/Tt: Inspiratory and expiratory time ratio
VT: Tidal volume
WBC: White blood cell count
